# Rare case of skeletal third class in a subject suffering from Solitary Median Maxillary Central Incisor syndrome (SMMCI) associated to panhypopituitarism

**DOI:** 10.1186/s13005-021-00300-3

**Published:** 2021-11-23

**Authors:** Alessandro Nota, Shideh Ehsani, Laura Pittari, Giorgio Gastaldi, Simona Tecco

**Affiliations:** Dental School and Postgraduate School of Orthodontics, Vita-Salute San Raffaele University and IRCCS San Raffaele Hospital, via Olgettina 58, Milan, Italy

**Keywords:** Panhypopituitarism, Dentistry, Hormonal deficiency, Congenital syndromes, Children disease, Dental anomalies, Pituitary gland, SMMCI, Interceptive orthodontics

## Abstract

**Background:**

The median solitary maxillary central incisor syndrome (SMMCI) is a rare malformative syndrome consisting of multiple defects, mainly found on the body midline. It can be correlated to the etiopathological and phenotypic pattern of panhypopituitarism. This case-report describes the rare case of a patient suffering from SMMCI and panhypopituitarism, showing an unusual craniofacial morphology.

**Case presentation:**

From the cephalometric analysis, a skeletal class III was identified (despite the other cases described in literature described as skeletal class II), derived from hypomaxillia and mandibular protrusion. A convex lip profile, with tendency to mandibular hyper-divergency, airway patency, anterior and posterior cross-bite were observed. At the clinical examination, a maxillary cant was evident on the frontal plane that appeared asymmetric, with the prevalence of the third lower part of the face. There were some dysmorphic signs such as: small nose, rectilinear eyelid line and reduced interocular distance.

**Conclusions:**

The present clinical case shows how, despite the literature, SMMCI can be associated with a III skeletal class, with maxillary hypoplasia and mandibular protrusion. The interdisciplinary collaboration between dentist and pediatrician is therefore important for the early interception of the malocclusions associated with these syndromes.

## Background

The median solitary maxillary central incisor syndrome (SMMCI) is a rare malformative syndrome consisting of multiple defects, mainly found on the body midline. Thus, the clinical finding of the presence of a single central incisor can be an isolated trait or be only an aspect of the SMMCI syndrome that includes other anomalies such as: intellectual disability, congenital heart disease, microcephaly, convergent strabismus, esophageal and duodenal problems, cervical atresia, cervical hemivertebrae, cervical dermoid, scoliosis, absent kidney, micropenis and ambiguous genitalia [1, 2]. In addition, it can also suggest the presence of holoprosencephaly [3], a complex brain malformation affecting both the forebrain and the face, resulting in various neurological deficits and facial defects [1].

The SMMCI is correlated to the etiopathological and phenotypic pattern of hypopituitarism, defined as a deficiency of one or more of the hormones produced by the pituitary gland. It can be attributed to a hypothalamus pathology, affecting the production of trophic hormones that act on the pituitary gland, or to a direct pathology of the pituitary gland itself [4]. The lack of pituitary hormones causes a reduction in the normal functionality of the peripheral endocrine glands, such as: thyroid, adrenal, gonads (ovaries in females and testicles in males), a deficiency of growth hormone (GH) and prolactin (very similar to GH as composition) [5]. A study conducted in 2001 in northern Spain reported an incidence of hypopituitarism of 45.5 people per 100,000, with 4.2 new cases per year [6]. The diagnosis is made through multiple hormonal tests and the search for the responsible pituitary lesion. About its clinical presentation, the most frequent deficit is about the growth hormone, which occurs with short stature and a growth speed reduction, associated with alteration of the body constitution (reduction in lean body mass and increase in fat mass) and craniofacial anomalies such as: splanchnocranium hypoplasia, frontal drafts, saddle nose, lip/palatal cleft.

In previous case reports [7, 8], and in a clinical study by Kjaer et al. [9] it was observed that patients with SMMCI, compared to the normal craniofacial parameters, have anterior cranial base and mandibular hypoplasia and frequently a skeletal class II.

In this scenario, the present case-report describes the apparently rare occurrence of severe skeletal class III in a subject suffering from SMMCI and panhypopituitarism.

## Case presentation

### Introduction of the clinical case

**Fig. 1:**
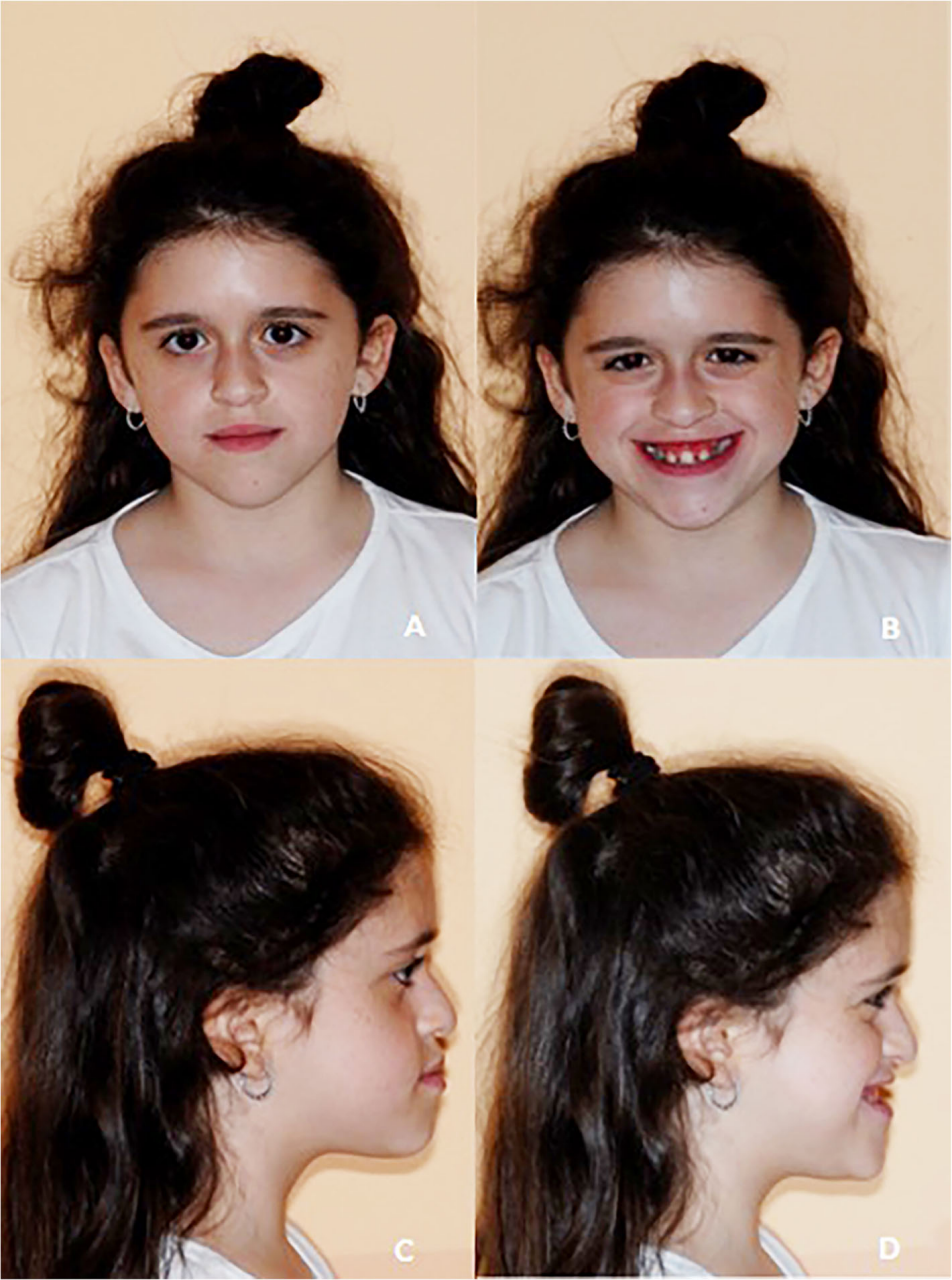
**a** Frontal extraoral photo; **b** Frontal extraoral photo with smile, **c** Lateral extraoral photo, **d** Lateral extraoral photo with smile

**Fig. 2:**
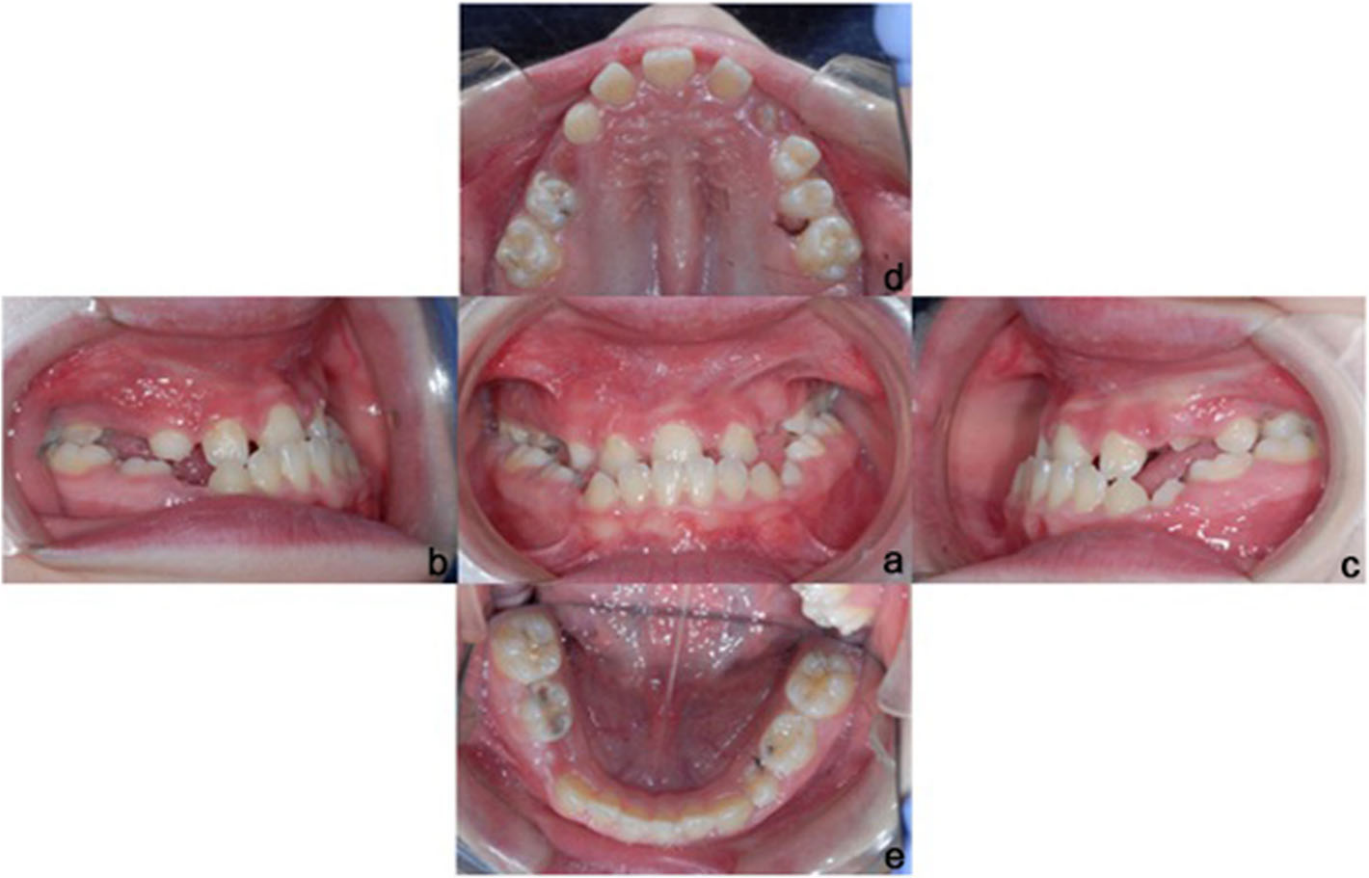
**a** Frontal intraoral photo, **b** Right intraoral photo, **c** Left intraoral photo, **d** Upper intraoral photo, **e** Lower intraoral photo

The patient came to our attention at the San Raffaele Dental Department (Milan, Italy) at the age of 11 years. During extraoral examination, a typical syndromic pattern was observed, due to an altered growth, concave profile, and short stature; no changes were observed that could lead to inflammatory diseases (Fig. 1a, b, c). At the intraoral examination, a dental class III was diagnosed, with mixed dentition, dental crowding and a single upper central incisor; in addition, teeth 5.4, 7.5 and 8.5 were affected by carious lesions and deciduous root residues were found in the arches. Oral hygiene was good (Fig. 2a, b, c, d, e). X-Ray investigations were requested to complete accurate craniofacial and orthodontic diagnosis (orthopantomography and lateral skull radiograph).

### Medical History

She was born with normal delivery at the 41st week of pregnancy, with birth weight 3480 g, length 50 cm (50th and 25-50th centile, respectively), from both parents of Caucasian race and normal stature. In the first hours of life, she had poor reactivity, feeding difficulties and slight signs of dyspnea for which she was subjected to oxygen therapy; for the detection of severe hypoglycaemia, she was infused with glucose solution. These problems persisted until the fifth day, with transient renal failure treated with Dopamine for 36 h. Upon examination of thyroid function, she reported non-measurable plasma TSH and FSH values. Therefore, in the suspicion of hypopituitarism, hormonal dosages were carried out confirming the diagnosis (GH <0.1ng / ml; cortisol 0.1 mg / dl; ACTH 13.7pg / ml; LH 0.1mUI / ml, FSH 0.2mUI / ml). MRI of the brain documented pituitary hypoplasia, excluding the possible presence of holoprosencephaly. Thus, hydrocortisone and L-Thyroxine therapy was started on day 5, with the presence of electrical crises treated with phenobarbital. On the eighth day, the patient was in good general condition, reactive pink, facies sui generis, saddle nose, low implant of the auricles.

From the following investigations it resulted:

- TSH: Insufficient, but it became progressively adequate after L-thyroxine therapy.
- GH: Insufficient, with IGF1 <25ng / ml; thus, she was treated with biosynthetic growth hormone therapy (0.1 mg / day).
- Water balance: adequate; urinary electrolytes, plasma and urinary osmolarity, and ADH dosage were then evaluated for suspected diabetes insipidus but resulted in the normal range.
- Neurological evaluation: following therapy with phenobarbital, EEG was normal; to be monitored; auditory and visual evoked potentials were in the norm.
- Cardiological evaluation: ECG was in the norm for age; one month after birth the echocardiogram showed patency of the oval foramen, to be monitored.
- Nutrition: during hospitalization meals were made with breast milk + milk adapted with bottles and SNG, adapting the quantity to the absence of weight increase, observed one month after birth. Subsequently the patient resumed feeding spontaneously, showing a constant weight increase (85-100kcl / kg / day).
- Eye examination: fundus oculi examination of both eyes posterior poles was apparently normal (right optic papilla slightly smaller than left one).
- Neuropediatric consultancy: the patient showed a head with normal morphology and fractional anisotropy (FA) large and flat, associated with minimal nonspecific dysmorphic signs (small nose, rectilinear eyelid lines without eyelashes), and no cranial nerve deficits. Eyeballs were on the axis, gaze following. She showed valid crying, normal tone and tropism, symmetrical active motility. When she was prone, she cleared the airways. Globally there was an age-appropriate neurological development.

The diagnosis of panhypopituitarism was then confirmed.

### Genetic Tests

The molecular analysis was carried out by new generation sequencing (NGS) of the candidate genes for panhypopituitarism (PROP1, POU1F1, GH1, GHRH, GHRHR, HESX1, PROKR2). In NGS genomic DNA was extracted from a peripheral blood sample with automated procedure using the Genecatcher kit (Invitrogen, USA). DNA was quantified with an ultrasensitive fluorescent nucleic acid stain (Quant-iT™ PicoGreen ® dsDNA Reagent and Kits, Invitrogen, USA). DNA library preparation was performed following the protocol of the TruSeq Custom Amplicon Library Preparation Kit (Illumina, USA) and hybridized with oligonucleotides probes that cover genomic regions of interest. The oligo custom panel design allows to evaluate all the exon regions and the exon/intron junctions of the genes studied. Hybridized DNA libraries were then sequenced using the sequencing by synthesis methodology (Illumina, USA) on a MiSeq sequencer. Sequencing data were analyzed with an informatics pipeline developed in house. Sequence variants were annotated using both an in house database and public database such as annovar and clineff. This test showed 100 % coverage of the portions examined with a minimum number of readings for each base not inferior than 20, identifying no significant alterations in the coding sequences of the examined genes.

### Clinical and X-ray examination

At the clinical extraoral examination, a maxillary cant was observable on the frontal plane that appeared asymmetric, with the prevalence of the third lower part of the face, due to the hypo-growth of the maxillary and upper third. In the face there were some dysmorphic signs such as: small nose, rectilinear eyelid line and reduced interocular distance. The mandible appeared prognathic on the sagittal plane, with a concave facial profile and no temporomandibular joint symptoms were observed [10].

Intraoral examination revealed molar and canine Angle class III with ogival palatum, mixed dentition, dental crowding and a single upper central incisor. There was a posterior bilateral crossbite and anterior cross-bite (inversion of the anterior relationship), with an accentuated Spee-curve.

**Fig. 3:**
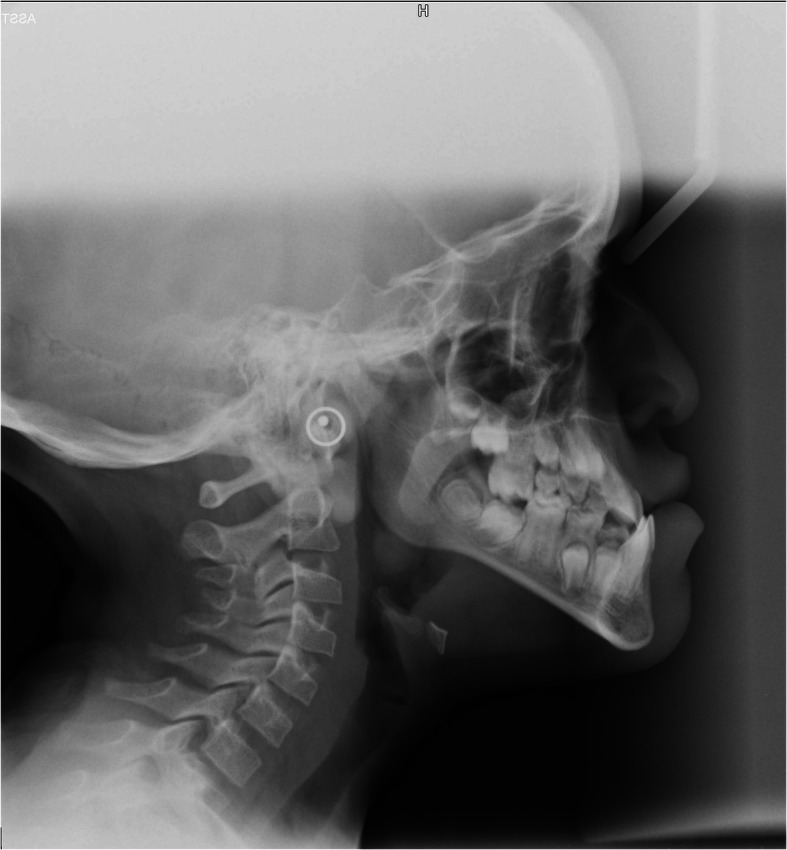
Lateral radiograph

From the lateral skull radiograph and its cephalometric analysis (Table 1), a hypoplasia of the sella turcica was observed and a skeletal class III was identified, derived from hypomaxillia and mandibular forward position, with convex lip profile, tendency to hyper-divergency, with growth by post-rotation, cervical vertebral maturation (CVM) at stage CS2, airway patency, anterior crossbite. An upper and lower incisal proclination was also detected (Fig. 3). No familiarity for skeletal class III was reported.

**Table 1:** Principal cephalometric measurements

Parameter	Values
SNA	82.5°
SNB	88.7°
ANB	-6.2°
Ar-Go-Gn	132.8°
Ar-Go-N	56.5°
SN^GoGn	33.9°
ANS-PNS^GoGn	29.5°
Is^ANS-PNS	110.6°
Ii^GoGn	85.9°
Wits	-19.2 mm

**Fig. 4:**
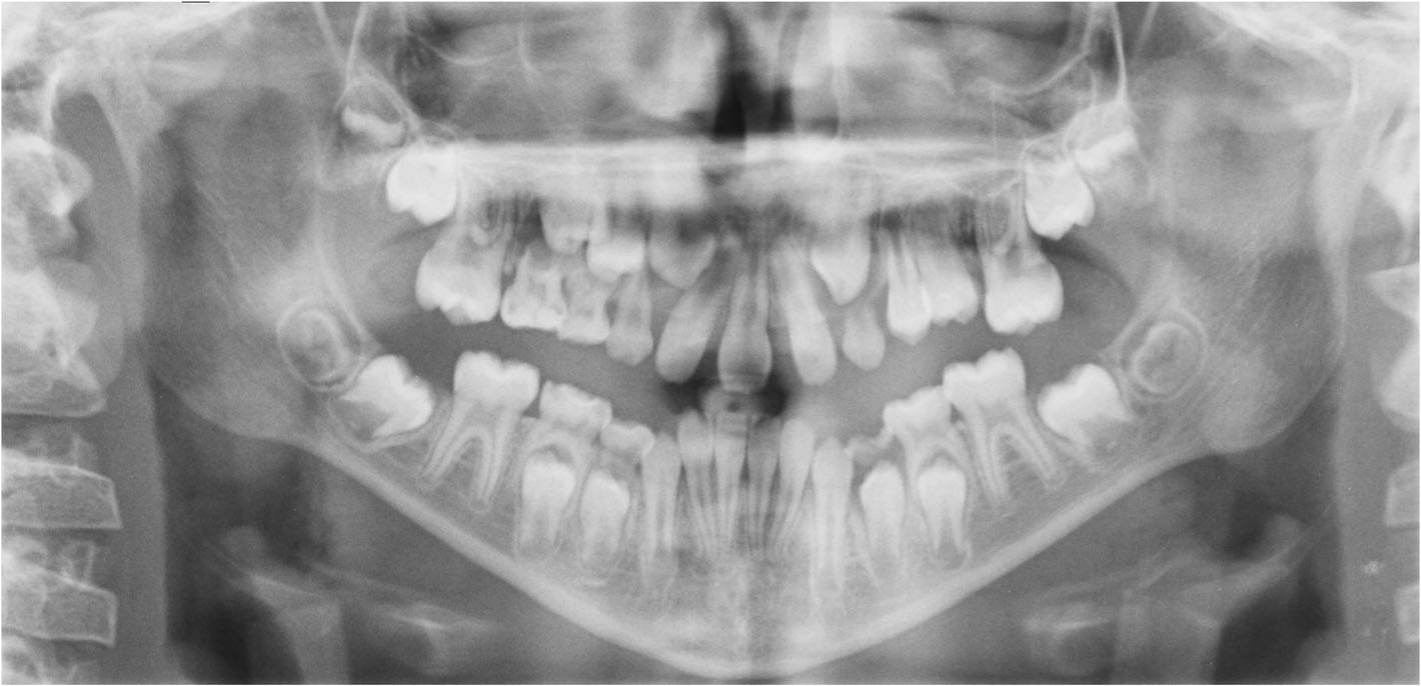
Orthopantomography

Orthopantomography, evidenced a complete asymmetric eruption of teeth 2.4 and 2.5, compared to 1.4 and 1.5, still in eruption. It also evidenced the agenesis of a central incisor (not easily determined if the 1.1 or 2.1). Correctly erupted first molars, the presence of erupting second molars, and initial forming third molars were observed (Fig. 4).

## Discussion

SMMCI is a rare anomaly that can present itself as an isolated finding or may be associated with systemic abnormalities. As the literature suggests this syndrome is characterized by a skeletal class II with hypoplastic and post-rotated maxilla, and a retrognathic jaw [9]. Differently from what previously reported in literature, an analysis of the radiographic exams performed in the present clinical case revealed a skeletal class III instead, with a tendency to hyper-divergency due to growth in mandibular postrotation, upper and lower incisor proclination and convex lip profile.

The intraoral examination confirmed the presence of a single medially positioned upper central incisor with mixed dentition. By the medical analyses, the patient resulted therefore suffering from panhypopituitarism and being treated with growth hormone, presenting concomitant SMMCI with exclusion of the co-presence of holoprosencephaly (as evident from postnatal MRI where only a reduced pituitary was highlighted).

SMMCI was initially reported by Scott who described a girl with the presence of a single maxillary central incisor placed in the median position, considering it as an isolated observation [11]. Another case of SMMCI was verified by Fulstow [12], who reported in addition to the single central incisor, short stature, congenital heart disease, microcephaly and scoliosis. The etiology is uncertain but some studies such as those conducted by Nanni et al. and Garavelli et al. have shown, following DNA sequencing, mutations of the Sonic Hedgehog (SHH) gene both in SMMCI syndrome and in holoprosencephaly frameworks [4, 13]. The diagnosis of SMMCI can be made at the age of eight months, but also at birth and even prenatally at the 18th-22nd week thanks to the mid-trimester routine ultrasound. SMMCI-related malformations result from unknown factors operating in the womb around the 35th or 38th day of pregnancy [14]. According to Yassin the presence of a single incisor instead of the two central incisors is given by the fusion of two neighboring teeth or the agenesis of a dental germ [15]. SMMCI may however be associated with other systemic disorders such as autosomal dominant holoprosencephaly, growth retardation and midline developmental defects, as the case shown in this report (except for the holoprosencephaly). Holoprosencephaly appears to be determined by the mutation of the SHH gene, as reported by Bertolacini [7]. In our clinical case this gene was not considered in genetic analysis due to the absence of holoprosencephaly, which was shown to be associated with the gene. Becktor et al. [8] assessed the intermaxillary suture, the single central incisor eruption pattern and upper jaw growth in a group of patients with SMMCI. The sample of Bertolacini’s work consisted of 11 patients with SMMCI, who underwent radiological investigations such as orthopantomography and teleradiography of the cranium in antero-posterior and latero-lateral projection. From the radiographic analysis, the presence of anomalies in the anterior portion of the incisor foramen on the median maxillary suture was observed, not however affecting the horizontal and vertical growth of the maxilla [7]. Analyzing 10 patients aged 8 to 17 years who presented with SMMCI, Kjaer et al. examined the clinical features and craniofacial morphology of this group of patients. Intraoral and extraoral photographs, lateral skull radiograph, orthopantomography and study models were analyzed. The results of the study showed that the craniofacial morphology of nine girls with SMMCI compared to the normal craniofacial parameters showed hypoplasia of the anterior cranial base, a hypoplastic and post-rotated maxilla, and a retrognathic and post-moved jaw, as well as morphological changes in the turcica; in addition, this group of patients had features such as: nasal obstruction, septal deviation, absence of frenulum of the upper lip and incomplete mid-palatal suture [9].

In the present clinical case report the patient shows a III skeletal class, unlike the literature tendency, with signs of manifest SMMCI related to panhypopituitarism. Cho et al. suggested that early detection of SMMCI is extremely important as the patient may present other severe congenital malformations in the evolution of the syndrome [16]. A limitation of this study is the absence of analysis of the SHH gene, however this occurred due to the absence of holoprosencephaly, which was shown to be associated with the gene. However, it has been found in the literature that, in a small percentage of cases, the gene mutation was observed associated with hypopituitarism in the absence of holoprosencephaly [17]. Furthermore, a Whole Exome Sequencing (WES) was not performed, so the genetic background of hypopituitarism cannot be completely excluded. In this complex syndromic pattern, management and treatment are complex and delicate. Above all, the psychological impact that this syndrome has on the patient and how the clinician can improve it, should be evaluated. Interdisciplinarity is fundamental for a correct management of the case and it should be considered that as a third skeletal class can be associated with the syndrome, an early orthodontic evaluation for a proper planning of the treatment timing is thus encouraged.

## Conclusions

The present clinical case shows how, despite the literature, SMMCI can be associated with a III skeletal class, with maxillary hypoplasia and mandibular protrusion. Although there is the lack of SHH gene mutation and the absence of holoprosencephaly, the patient is affected by SMMCI with concomitant pan-hypopituitarism. We can therefore observe how the presence of SMMCI should not be considered as a simple dental anomaly, since it can be associated with other clinical features and craniofacial malformations. Interdisciplinarity is therefore important for the early evaluation of the malocclusions associated with these syndromes.

## Data Availability

The data that support the findings of this study are available from the archive of the Vita-Salute San Raffaele University, but restrictions apply to the availability of these data, which were used under permission and consent for the current study, and so are not publicly available. Data are however available from the authors upon reasonable request and with permission of the patients and the Ethic Committee of the Vita-Salute San Raffaele University.

## Abbreviations

SMMCI: Solitary median maxillary central incisor syndrome
TSH: Thyroid stimulating hormone
GH: Growth hormone
IGF1: Insulin-like growth factor 1
ADH: Antidiuretic hormone
FSH: Follicle Stimulating Hormone
ACTH: Adrenocorticotropic hormone
LH: Luteinizing hormone
MRI: Magnetic Resonance Imaging
ECG: Electrocardiogram
EEG: Electroencephalogram
NGS: New generation sequencing
CVM: Cervical vertebral maturation
SHH: Sonic Hedgehog gene
FA: Fractional anisotropy
